# Effect of goalkeepers’ offensive participation on team performance in the women Spanish La Liga: a multinomial logistic regression analysis

**DOI:** 10.5114/biolsport.2024.125592

**Published:** 2023-05-25

**Authors:** Claudio A. Casal, Joseph A. Stone, Iyán Iván-Baragaño, José L. Losada

**Affiliations:** 1 Department of Science of Physical Activity and Sport, Catholic University of Valencia, San Vte Mártir, Valencia, Spain; 2 Academy of Sport and Physical Activity, Sheffield Hallam University, United Kingdom; 3 Faculty of Sport Sciences, Universidad Europea de Madrid, Madrid, Spain; 4 Department of Social Psychology and Quantitative Psychology, University of Barcelona, Barcelona, Spain

**Keywords:** Female football, Observational methodology, Goalkeeper’s distribution, Match analysis, Key performance indicators, Multinomial logistic regression

## Abstract

This study aimed to examine the effect of goalkeeper distribution on offensive team performance, during the 2018/2019 and 2019–2020 seasons of the Women Spanish La Liga. A total of 10,868 distributions, during 376 matches were analyzed by systematic observation. Two UEFA PRO coaches designed an ad hoc observation instrument “GOALDFOOT” and one observer coded the data after a training process. An intra-observer reliability kappa index of 0.94 was established. Results show how the offensive effectiveness of the goalkeepers was similar to outfield players, with 0.4% of possessions ending in a goal, 2.2% ending in an attempt on goal, with 79.4% ending unsuccessfully. The goalkeeper lost possession from their distribution 32.5% of the time. Multivariate analysis identified several predictors of goalkeepers’ distributions. The results show that teams classified in the middle zone of the final classification of the regular league had 1.2 times more probability of being successful compared with the lowest ranked teams (p < 0.05). Goalkeeper’s distribution beginning during Open play after a transition, represented an increase success rate of almost 3 times compared to being performed from a free kick (p < 0.05). Passes from outfield players to a goalkeeper made from distant zones to the own goal, decreased the probability of success (p < 0.001). The pitch location of the distribution outcome near to the opponent goal offered the best probability of success. In conclusion, the most effective offensive sequences occur with dynamic transitions initiated with short passes. This information can provide coaches and players with insights to improve the offensive performance of goalkeepers.

## INTRODUCTION

The specific position of afootball goalkeeper has undergone a remarkable evolution in recent times. A key factor in this transformation, is the regulatory change that prevents the goalkeeper from receiving the ball from a teammate with their hands (1992). This rule allows opponents to press the ball, while the goalkeeper is in possession of the ball, and forces the goalkeeper to play the ball with their feet, requiring greater technical mastery [1, 2]. The goalkeeper has ceased to be solely responsible for the defence of the goal, to have a much more active role, both in the defensive and offensive phases of open play. For example, Pérez-Muñoz et al. [3] demonstrated the percentage of offensive technical actions represents 64.72% of goalkeeper actions during a match. Furthermore, research has identified the predominant technical-tactical actions in football goalkeepers in the Spanish La Liga are foot controls, short passes and goal kicks [1, 4].

Offensively, the football goalkeeper has become another outfield player, starting, and giving continuity to the game, offering a pass option to the player with the ball and becoming a fundamental part in determining the team’s offensive game model. Defensively, in addition to being responsible for defending the goal, on many occasions, they are also required to play away from it and to become another defender in defensive transitions [5, 6]. Consequently, this role has increased its relevance in today’s football, and it would be interesting to know how the goalkeeper participates in the offensive phase of the team and their importance in its performance. However, scientific knowledge, to date, is scant. In the limited research, there has been investigations into the number and type of offensive and defensive technical actions performed by goalkeepers during matches [3] and a comparative analysis of the technical-tactical actions of goalkeepers based on the competitive category and the location of the match, which reported significant differences in both cases [1]. The only study like the one we propose here, is that of Seaton and Campos [7], who found significant differences in the type of distribution and the success of these, between goalkeepers of teams of different skill levels. But research to date has not attempted to establish a direct relationship between the characteristics of the goalkeeper’s offensive participation and the team’s offensive performance.

If we focus on women’s football, the number of works published to date is even lower. We have only found the work of Sainz de Baranda et al. [6], who carried out an analysis of the technical-tactical actions of the goalkeepers of the Women’s FIFA World Cup 2011, to determine the relationship between these actions and the qualifying results of their respective teams, concluding that the goalkeepers of the teams that surpassed the group stages have a greater offensive participation, as well as a greater number of passes completed successfully in different areas of the field. While the goalkeepers of the unclassified teams show greater defensive actions, such as saves inside the penalty area, foot saves and failed punches. Therefore, we have not found any previous work that analyses whether there is a direct relationship between the type of distribution made by the goalkeeper and the team’s offensive performance in any women’s football competition.

Consequently, due to the non-existent scientific evidence on the influence of the offensive participation of the goalkeeper in the result of the offensive phase of the team in football, we propose the objectives of describing the characteristics of the offensive sequences in the offensive participation of the goalkeeper in the 2018/19 and 2019/20 seasons of the Women Spanish La Liga, to identify the Key Performance Indicators (KPI) in these game situations and, finally, to understand how these indicators can predict the final result of offensive sequences.

For this, we used an observational methodology, since, in team sports and specifically in football, notational analysis through systematic observation is an effective and objective instrument to collect information and identify the most relevant events that occur in them. As Carling et al. [8] highlights when affirming that match analysis has taken a transcendental role in sports. In many cases, observation is the only scientific method that allows data collection directly from the participants in competition without disturbing their action. This observation is typically performed by recording the data through an ad hoc observation instrument while participants act in their natural context [9].

The results will offer information on the characteristics of the distributions with the highest probability of offensive success, which players and coaches can use to apply it to their teams, to aid with performance improvement.

## MATERIALS AND METHODS

### Design and sample

The specific design corresponding to this systematic observation, according to Anguera et al. [10], was a nomothetic/follow up/multi-dimensional (N/F/M) design. Moreover, the recording used an intra-sessional follow-up observation (frame-by-frame analysis of different matches) and was captured, post event, using the ad hoc observation instrument. Data analyzed is of type IV [11].

All teams (n = 18) and 376 games from the 2018/19 and 2019/20 seasons of La Liga Iberdrola were analyzed, resulting in 10,868 goalkeepers’ distributions, cropped from full game footage obtained from InStat Ltd (http://instatsport.com). The recording of the information was carried out respecting the behavior spontaneity of the players and in their natural environment. According to the Belmont Report [12], the use of public images for research purpose does not require informed consent or the approval of an ethical committee.

### Observation instrument

**FIG. 1:**
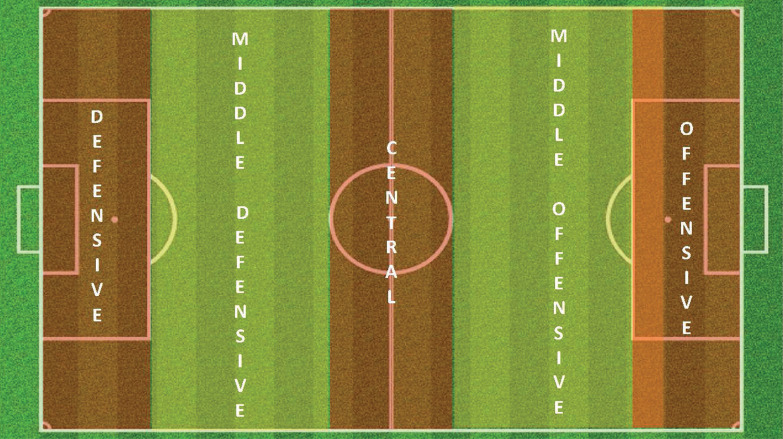


Anguera et al. [13] guidance was followed for the creation of the observation instrument. First, a hierarchical range of behavior units was established, which was implemented through the adoption of basic criteria for behavior segmentation. The creation of the observation instrument was based on the following pillars: i) a previous theoretical framework; ii) criteria and categories compiled empirically in other observational studies; iii) and, finally, novel criteria that were tested in this work. The methodological steps implemented were the following: First, the problem was identified, and an expert scientific group was formed, comprising of two academic (with PhDs in Physical Activity and Sports Sciences) and UEFA PRO coaches, with more than ten years of experience in observational methodology and performance football analysis. After consulting the theoretical framework and empirical evidence, a first postevent exploratory observation was made. Then, and after a discussion by the group of experts, the problem was divided into smaller units. Subsequently, an ad hoc observation instrument, denominated GOALDFOOT (Table 1), consisting of field format and category systems, was created, and tested in order to find weaknesses in the instrument itself. Then, after further discussion by the group of experts, the observation instrument was readjusted. Finally, the post-event viewing was carried out, to finalize the implementation of the observation instrument. The field format was divided into five zones parallel to the goal [14], (Figure 1).

**TABLE 1:** Criteria, categories, and codes to observational instrument

Criteria	Category	Code
Location L	**Home**: The observed team plays at home	HM
	**Away**: The observed team plays away from home	AW

Team quality TQ	**Best teams:** The best five ranked teams at the end of the regular league	G1
	**Medium teams:** The six teams classified in the middle zone of the final classification of the regular league	G2
	**Bottom teams:** The five lowest ranked teams at the end of the regular league	G3

Time T	**0–15 Minutes:** The goalkeeper distribution started within 0–15 minutes of the match time	0–15
	**15–30 Minutes:** The goalkeeper distribution started within 16–30 minutes of the match time	15–30
	**30–45 Minutes:** The goalkeeper distribution started within 31 minutes – half time	30–45
	**45–60 Minutes:** The goalkeeper distribution started within 45–60 minutes of the match time	45–60
	**60–75 Minutes:** The goalkeeper distribution started within 61–75 minutes of the match time	60–75
	**75–90 Minutes:** The goalkeeper distribution started within 76 minutes – full time	75–90

Final Result FR	**Win:** The attacking team has scored more goals than the opponent and won the match	FW
	**Draw:** The attacking team has scored equal goals to the opponent and draw the match	FD
	**Loss:** The attacking team has scored fewer goals than the opponent and lost the match	FL

Match Status MS	**Winning:** The team in possession has scored more goals than the opposition and at the time of the goalkeeper distribution	WS
	**Drawing:** The team in possession has scored equal goals to the opposition at the time of the goalkeeper distribution or no goals had been scored	DR
	**Losing:** The team in possession has scored fewer goals than the opponent at the time of the goalkeeper distribution	LS

Distribution D	**Direct:** The goalkeeper distributes the ball to an attacking outfield player or an area of space in the middle or offensive locations of the pitch, the outfield player must have enough control over the ball to be able to have a deliberate influence on the ball’s subsequent direction	DR
	**Indirect:** The goalkeeper distributes the ball to a defensive outfield player in the defensive zones of the pitch, the outfield player must have enough control over the ball to be able to have a deliberate influence on the ball’s subsequent direction	ID

Distribution Type DT	**Goal Kick:** The distribution of play was started by the goalkeeper from a goal kick	GK
	**Free Kick:** The distribution of play was started by the goalkeeper from a free kick	FK
	**Open play to continue the possession:** The distribution of play was started by the goalkeeper from open play after a pass from a player from the same team	OP
	**Open play after transition:** The distribution of play is started by the goalkeeper from open play after the recovery of the ball and to start the offensive transition	OR

Distribution Zone DZ	**Inside the box:** The goalkeeper started the distribution inside the penalty area	IB
	**Outside the box:** The goalkeeper started the distribution outside the penalty area	OB

Defensive Pressure DP	**High Press:** A player from the opposing team is pressing the goalkeeper when they start the distribution	HG
	**Low Press:** The goalkeeper started the distribution without an opposition player near them	LW

Number of passes NP	**0:** No passes were completed, including the goalkeeper distribution, before an outcome was performed	0
	**1–3:** 1–3 passes were completed including the goalkeeper distribution before an outcome was performed	1–3
	**4–6:** 4–6 passes were completed including the goalkeeper distribution before an outcome was performed	4–6
	**> 6:** More than 6 complete passes occurred including the goalkeeper distribution before an outcome was > 6 performed

Pitch Location of Distribution PLD	**Defensive:** The ball is distributed by the goalkeeper into the defensive zone of the pitch	DF
	**Middle Defensive:** The ball is distributed by the goalkeeper into the middle defensive zone of the pitch	MD
	**Central:** The ball is distributed by the goalkeeper into the central zone of the pitch	CE
	**Middle Offensive:** The ball is distributed by the goalkeeper into the middle offensive zone of the pitch	MO
	**Offensive:** The ball is distributed by the goalkeeper into the offensive zone of the pitch	OF

Pitch Location of Outcome PLO	**Defensive:** The outcome of play is performed in the defensive zone of the pitch	DF
	**Middle Defensive:** The outcome of play is performed in the middle defensive zone of the pitch	MD
	**Central:** The outcome of play is performed in the central zone of the pitch	CE
	**Middle Offensive:** The outcome of play is performed in the middle offensive zone of the pitch	MO
	**Offensive:** The outcome of play is performed in the offensive zone of the pitch	OF

Pitch Zone of First Pass by Outfield Play PZFPO	**Ø:** Goalkeeper does not receive the ball by an outfield player	**Ø**
	**Defensive:** The ball is passed by the outfield player to the goalkeeper into the defensive zone of the pitch	DF
	**Middle Defensive:** The ball is passed by the outfield player to the goalkeeper into the middle defensive zone of the pitch	MD
	**Central:** The ball is passed by the outfield player to the goalkeeper into the central zone of the pitch	CE
	**Middle Offensive:** The ball is passed by the outfield player to the goalkeeper into the middle offensive zone of the pitch	MO
	**Offensive:** The ball is passed by the outfield player to the goalkeeper into the offensive zone of the pitch	OF

Outcome OUT	**Goal:** When the whole of the ball crosses over the line, between the goal posts and under the crossbar, provided no offence has been committed by the scoring team. The referee awarded a goal	GO
	**Attempt ON Target:** An attempt on goal by the attacking team that were heading towards the goal which was saved by the goalkeeper or blocked by a defensive player of the opposing team	AO
	**Attempt OFF Target:** An attempt by the attacking team which was not directed between the dimensions of the goal including hitting the crossbar or goal posts	AF
	**Set-play:** A set piece was awarded to the attacking team in the form of a free kick, corner, penalty kick or SP throw-in
	**Loss of Possession:** The attacking team lost possession of the ball through the ball going out of the dimensions of the pitch or an opposing team player regaining possession of the ball, with enough control to have a deliberate influence over the ball’s subsequent direction	LP
	**Goalkeeper Loss of Possession:** The attacking team lost possession of the ball through the ball going out of the dimensions of the pitch or directly to an opposition player directly from the goalkeeper’s distribution of the ball	LG
	**Returned to Goalkeeper:** The team with possession pass the ball back to the goalkeeper. The goalkeeper has enough control over the ball to have a deliberate influence over the ball’s subsequent direction	RG

### Procedure and reliability

Data were coded by one observer and prior to the coding process, and to reduce intra-observer variability, eight training sessions, lasting two hours, were carried out following the Losada and Manolov [15] criteria and applying the criterion of consensual agreement [16] among the observer and the principal investigator, so that recording was only done when agreement was produced. A total of 857 distributions were analyzed in the training sessions. An intra-observer reliability test was conducted through reassessment of 1,087 goalkeeper distributions (10%), [17] randomly selected, four weeks after the initial analysis [18]. Cohens’s Kappa coefficient calculation [19] was used to quantify the intra-observer reliability of the data collected by the researcher. Reliability of each category is presented in Table 2, with the number of passes presenting the lowest value (0.85), considered excellent according to Fleiss et al. [20] scale.

**TABLE 2:** Intra-observer reliability values for notational analysis data quantified using a Cohen’s Kappa calculation

Criteria	Intra-rater value
Time	1.00
Distribution	1.00
Distribution Type	1.00
Distribution Zone	0.94
Defensive Pressure	0.89
Number of Passes	0.85
Pitch Location Distribution	0.96
Pitch Location Outcome	0.87
Pitch Zone of First Pass by Outfield Play	0.92
Outcome	0.98

**KTotal**	**0.94**

### Data analysis

In accordance with the aims of the study, both descriptive (frequency distribution tables) and inferential statistics (bivariate and multivariate analysis) were used in the analysis. The bivariate analysis (Pearson’s χ^2^) examined the association between the outcome and explanatory variables and the effect size was calculated from the contingency coefficient. The effect size was calculated and described as small (ES = 0.10), medium (ES = 0.30) or large (ES > 0.50) [21]. For multivariate statistical analysis, first, we recoded the Outcome into three new criteria: Successful (goal, attempt on and off target), unsuccessful (loss of possession, goalkeeper loss of possession) and possession continued (set-play). All distribution, which resulted in a return to goalkeeper were excluded (1,011), as this was deemed a neutral outcome, and began a new goalkeeper distribution, therefore resulting in the final analysis of 9,857 distributions. Multinomial logistic regression analysis was then used to examine which factors significantly influenced the outcome sequences involving the goalkeeper. Our reference category in the regression analysis was the unsuccessful outcome, and the results of the multinomial logistic regression analysis are presented as odds ratios. We also calculated the effect size [22] based on the coefficient of determination R^2^_N_. R program (v.3.4.1) using “nnet” library was used to run all analyses, and the level of significance for each performance indicator was set at 5% (p < 0.05) as usual in comparable scientific studies [23].

## RESULTS

### Descriptive and bivariate analysis

A total of 10,868 goalkeeper distributions were analyzed within the study, with an average of 28.9 per game, of which 0.4% ended in a goal, 2.2% ended with an attempt and in 79.4% of the occasions there was a loss of possession. The goalkeeper loss possession 32.5% of the time. Table 3 displays the results of the descriptive and bivariate analysis of the offensive play in which there was an offensive intervention by the goalkeeper. The best (p < 0.001), win (p < 0.001) and winning teams (p = 0.005) achieved more exits than the rest of the teams. There were significant differences (p < 0.001) between direct and indirect distributions. Indirect distributions were more successful than the direct distribution which usually ended with goalkeeper loss of possession (92.6%). Goalkeeper distributions were most common from Open play (38%). The offensive sequences with 4–6 passes were the most successful (p < 0.001). The pitch location distribution resulting in the most unsuccessful outcome was the offensive zone, with the middle defensive zone being the most successful (p < 0.001).

**TABLE 3:** Absolute frequencies, percentage occurrence of total distribution and association with outcome

	Outcome								

	EX			NEX		CP		χ^2^	

	Attempt Off Target	Attempt On Target	Goal	GK Loss of Possession	Loss of Possession	Set play	Returned to Goalkeeper	*P*-value	*ES*
**Location**								0.174	---
Home	70 (53.4%)	51 (46.8%)	29 (64.4%)	1529 (47.7%)	2757 (50.8%)	441 (47.0%)	490 (48.5%)		
Away	61 (46.6%)	58 (53.2%)	16 (35.6%)	1678 (52.3%)	2670 (49.2%)	497 (53.0%)	521 (51.5%)		

**Team Quality**								< 0.001	0.08
Best teams	79 (60.3%)	59 (54.1%)	29 (64.4%)	892 (27.8%)	2208 (40.7%)	365 (38.9%)	488 (48.3%)		
Medium teams	28 (21.4%)	36 (33.0%)	12 (26.7%)	1246 (38.9%)	1822 (33.6%)	349 (37.2%)	317 (31.4)		
Bottom teams	24 (18.3%)	14 (12.8%)	4 (8.9%)	1069 (33.3%)	1397 (25.7%)	224 (23.9%)	206 (20.4%)		

**Time**								0.579	---
0–15	17 (13%)	19 (17.4%)	12 (26.7%)	524 (16.3%)	1093 (20.1%)	174 (18.6%)	202 (20.0%)		
16–30	19 (14.5%)	21 (19.3%)	6 (13.3%)	474 (14.8%)	926 (17.1%)	171 (18.2%)	193 (19.1%)		
31–HT	22 (16.8%)	12 (11%)	4 (8.9%)	483 (15.1%)	857 (15.8%)	156 (16.6%)	202 (20.0%)		
46–60	22 (16.8%)	14 (12.8%)	10 (22.2%)	511 (15.9%)	864 (15.9%)	131 (14.0%)	159 (15.7%)		
61–75	24 (18.3%)	14 (12.8%)	9 (20.0%)	488 (15.2%)	814 (15.0%)	144 (15.4%)	134 (13.3%)		
76–FT	27 (20.6%)	29 (26.6%)	4 (8.9%)	727 (22.7%)	873 (16.1%)	162 (17.3%)	121 (12.0%)		

**Final Result**								< 0.001	0.050
Draw	30 (22.9%)	28 (25.7%)	3 (6.7%)	772 (24.1%)	1225 (22.6%)	213 (22.7%)	190 (18.8%)		
Loss	36 (27.5%)	31 (28.4%)	8 (17.8%)	1241 (38.7%)	2037 (37.5%)	337 (35.9%)	363 (35.9%)		
Win	65 (49.6%)	50 (45.9%)	34 (75.6%)	1194 (37.2%)	2165 (39.9%)	388 (41.4%)	458 (45.3%)		

**Match Status**								0.005	0.039
Drawing	55 (42.0%)	49 (45.0%)	18 (40.0%)	1505 (46.9%)	2577 (47.5%)	440 (46.9%)	435 (43.0%)		
Losing	28 (21.4%)	26 (23.9%)	5 (11.1%)	848 (26.4%)	1421 (26.2%)	249 (26.5%)	257 (25.4%)		
Winning	48 (36.6%)	34 (31.2%)	22 (48.9%)	854 (26.6%)	1429 (26.3%)	249 (26.5%)	319 (31.6%)		

**Distribution**								< 0.001	0.142
Direct	34 (26.0%)	36 (33.0%)	14 (31.1%)	2970 (92.6%)	1699 (31.3%)	313 (33.4%)	47 (4.6%)		
Indirect	97 (74.0%)	73 (67.0%)	31 (68.9%)	237 (7.4%)	3728 (68.7%)	625 (66.6%)	964 (95.4%)		

**Distribution Type**								< 0.001	0.074
Free Kick	4 (3.1%)	1 (0.9%)	1 (2.2%)	234 (7.3%)	243 (4.5%)	293.1%	38 (3.8%)		
Goal Kick	16 (12.2%)	18 (16.5%)	5 (11.1%)	947 (29.5%)	1343 (24.7%)	209 (22.3%)	261 (25.8%)		
OP	59 (45.0%)	50 (45.9%)	21 (46.7%)	1084 (33.8%)	2133 (39.3%)	397 (42.3%)	449 (44.4%)		
OR	52 (39.7%)	40 (36.7%)	18 (40.0%)	942 (29.4%)	1708 (31.5%)	303 (32.3%)	263 (26.0%)		

**Distribution zone**								0.208	---
Inside box	109 (83.2%)	89 (81.7%)	41 (91.1%)	2759 (86.0%)	4728 (87.1%)	830 (88.5%)	919 (90.9%)		
Outside box	22 (16.8%)	20 (18.3%)	4 (8.9%)	448 (14.0%)	699 (12.9%)	108 (11.5%)	92 (9.1%)		

**Defensive pressure**								0.06	---
High press	20 (15.3%)	15 (13.8%)	10 (22.2%)	912 (28.4%)	801 (14.8%)	155 (16.5%)	74 (7.3%)		
Low press	111 (84.7%)	94 (86.2%)	35 (77.8%)	2295 (71.6%)	4626 (85.2%)	783 (83.5%)	937 (92.7%)		

**Number of passes**								< 0.001	0.272
0	0 (0.0%)	0 (0.0%)	2 (4.4%)	3203 (99.9%)	7 (0.1%)	82 (8.7%)	2 (0.2%)		
1–3	39 (29.8%)	34 (31.2%)	12 (26.7%)	3 (0.1%)	3770 (69.5%)	591 (63.0%)	807 (79.8%)		
4–6	54 (41.2%)	34 (31.2%)	17 (37.8%)	0 (0.0%)	1136 (20.9%)	171 (18.2%)	161 (15.9%)		
> 6	38 (29.0%)	41 (37.6%)	14 (31.1%)	1 (0.0%)	514 (9.5%)	94 (10.0%)	41 (4.1%)		

**Pitch Location of Distribution**								< 0.001	0.213
Defensive	17 (13.0%)	10 (9.2%)	8 (17.8%)	0 (0.0%)	1066 (19.6%)	167 (17.8%)	403 (39.9%)		
MD	88 (67.2%)	66 (60.6%)	27 (60.0%)	11 (0.3%)	3079 (56.7%)	522 (55.7%)	590 (58.4%)		
Central	23 (17.6%)	27 (24.8%)	5 (11.1%)	3 (0.1%)	1100 (20.3%)	144 (15.4%)	16 (1.6%)		
MO	3 (2.3%)	5 (4.6%)	3 (6.7%)	0 (0.0%)	166 (3.1%)	23 (2.5%)	0 (0%)		
Offensive	0 (0.0%)	1 (0.9%)	2 (4.4%)	3193 (99.6%)	16 (0.3%)	82 (8.7%)	2 (0.2%)		

**Pitch Location of Outcome**								0.532	---
Defensive	0 (0.0%)	0 (0.0%)	0 (0.0%)	40 (1.2%)	40 (0.7%)	17 (1.8%)	950 (94%)		
MD	0 (0.0%)	0 (0.0%)	1 (2.2%)	611 (19.1%)	736 (13.6%)	214 (22.8%)	59 (5.8%)		
Central	0 (0.0%)	0 (0.0%)	2 (4.4%)	1916 (59.7%)	1742 (32.1%)	319 (34.0%)	1 (0.1%)		
MO	44 (33.6%)	28 (25.7%)	4 (8.9%)	603 (18.8%)	1983 (36.5%)	270 (28.8%)	0 (0.0%)		
Offensive	87 (66.4%)	81 (74.3%)	38 (84.4%)	37 (1.2%)	926 (17.1%)	118 (12.6%)	1 (0.1%)		

**Pitch Zone of First Pass by Outfield Play**								0.416	---
**Ø**	72 (55.0%)	59 (54.1%)	24 (53.3%)	2136 (66.6%)	3309 (61.0%)	542 (57.8%)	562 (55.6%)		
Defensive	10 (7.6%)	7 (6.4%)	5 (11.1%)	222 (6.9%)	375 (6.9%)	70 (7.5%)	81 (8%)		
MD	41 (31.3%)	39 (35.8%)	16 (35.6%)	795 (24.8%)	1594 (29.4%)	304 (32.4%)	339 (33.5%)		
Central	8 (6.1%)	4 (3.7%)	0 (0.0%)	54 (1.7%)	144 (2.7%)	22 (2.3%)	29 (2.9%)		
MO	0 (0.0%)	0 (0.0%)	0 (0.0%)	0 (0.0%)	5 (0.1%)	0 (0.0%)	0 (0.0%)		

EX: successful; NEX: unsuccessful; CP: continued possession; OP: Open play to continue the possession; OR: Open play after transition; MD: Middle defensive; MO: Middle offensive; ES: Effect Size calculated as contingency coefficient

### Multivariate analysis

Table 4 shows the results of the multinomial analysis comparing the unsuccessful results (NEX) with the successful ones (EX) and with continuing possession of the ball (CP). The model explained 87.66% of the changes in outcome of offensive sequences with goalkeeper distribution, suggesting that it is a good fit with the data. The accuracy of the test dataset was 0.17% higher compared to the training dataset, therefore we did not have an overfitting problem. The coefficient of determination R^2^_N_ has a small value of 0.165, according to the Cohen’s scale [21], (small, ES = 0.21–0.49; medium, ES = 0.50–0.70 or large, ES > 0.80).

**TABLE 4:** Multinomial logistic regression predicting to scoring, achieve scoring opportunity and continued possession vs. loss possession (Reference Category).

	Goalkeeper’s Distribution Outcome							

	CP				EX			

Predictor	*β*	*P*	Odds ratio	IC (95%)	*β*	*P*	Odds ratio	IC (95%)
**Team Quality**								
1	0.02	0.80	1.0249	0.84–1.24	0.26	0.19	1.30	0.87–1.93
2	0.18	0.04	1.2037	1.00–1.44	0.09	0.63	1.10	0.73–1.65
3^#^								

**Match Location**								
Home	0.16	0.06	0.85	0.73–0.97	0.16	0.24	0.85	0.64–1.11
Away^#^								

**Time**								
16–30	0.14	0.23	1.15	0.91–145	-0.02	0.90	0–97	0.62–1.52
31–HT	0.15	0.21	1.16	0.91–1.49	-0.20	0.39	0.81	0.50–1.30
46–60	-0.02	0.84	0.97	0.75–1.26	0.07	0.77	1.07	0.66–1.72
61–75	0.12	0.32	1.13	0.88–1.47	0.07	0.76	1.07	0.67–1.72
76–FT	0.16	0.19	1.18	0.91–1.52	0.39	0.09	1.48	0.93–2.34
0–15^#^								

**Final Result**								
Draw	0.09	0.39	1.09	0.88–1.36	0.22	0.32	1.24	0.80–1.93
Win	0.16	0.15	1.18	0.94–1.48	0.35	0.12	1.41	0.90–2.23
Loss^#^								

**Match Status**								
Drawing	-0.05	0.63	0.94	0.76–1.18	-0.06	0.76	0.93	0.59–1.46
Winning	-0.10	0.42	0.89	0.68–1.17	0.03	0.90	1.03	0.61–1.72
Lossing^#^								

**Distribution zone**								
Outside box	21.31	0.06	1.80e0+9	1.20e0+9–2.69e0+9	34.41	0.072	8.85e+14	5.06e0+14–1.55e+15
Inside box^#^								

**Dristribution Type**								
Goal Kick	0.17	0.40	1.19	0.78–1.82	0.59	0.21	1.80	0.70–4.60
Open Play Continue	-0.27	0.68	0.75	0.20–2.88	0.42	0.69	1.52	0.18–12.36
Open Play Transition	0.38	0.06	1.46	0.97–2.20	0.98	0.03	2.68	1.10–6.51
Free Kick^#^								

**Distribution**								
Indirect	-0.01	0.90	0.98	0.76–1.26	-0.33	0.20	0.71	0.43–1.19
Direct^#^								

**Nº passes**								
4–6	0.09	0.35	1.10	0.90–1.34	0.74	< 0.001	2.10	1.51–2.91
> 6	0.35	0.007	1.42	1.10–1.85	1.15	< 0.001	3.19	2.22–4.57
1–3^#^								

**Pitch Zone o First Pass by Outfield Play**								
Central	-0.04	0.88	0.96	0.55–1.67	0.09	0.84	1.09	0.46–2.59
Middle Defensive	0.08	0.60	1.08	0.81–1.45	-2.60	< 0.001	0.07	0.07–0.07
Middle Offensive	-27.70	< 0.001	9.25e-13	9.25e-13–9.25e-13	-24.92	< 0.001	1.50e-11	1.50e-11–1.50e-11
Defensive^#^								

**Pitch Location of Distribution**								
Central	0.01	0.95	1.01	0.71–1.43	0.47	0.17	1.60	0.81–3.15
Middle Defensive	0.09	0.41	1.09	0.88–1.34	0.08	0.71	1.08	0.71–1.65
Middel Offensive	0.21	0.47	1.23	0.70–2.15	0.19	0.70	1.20	0.47–35.50
Offensive	-0.55	0.58	0.57	0.08–4.13	1.24	0.30	3.46	0.34–35.50
Defensive^#^								

**Pitch Location of Outcome**								
Central	-0.88	0.003	0.42	0.23–0.74	8.42	< 0.001	4538.25	1374.93–14979.48
Middle Defensive	-0.38	0.19	0.68	0.38–1.20	8.70	< 0.001	6042.99	1246.18–29303.86
Middle Offensive	-1.14	< 0.001	0.32	0.18–0.57	11.80	< 0.001	133801.63	71905.97–248976.22
Offensive	-1.25	< 0.001	0.28	0.16–0.52	13.37	< 0.001	640716.10	345793.16–1.19e0+6
Defensive^#^

**Defensive Pressure**								
High	26.47	0.08	3.14e+11	2.13e+11–4.62e+11	13.25	0.10	565577.77	32459.43–98548.55
Low^#^								

# , Reference category; β, Beta coefficient; CI, Confidence interval; p < 0.05, p < 0.01, p < 0.001.

Compared to the bottom teams (3), the medium teams (2) were 1.2 times more likely to continue possession. The open play after transition achieved 2.7 more probability of success than distribution from free kicks. Increasing the number of passes in offensive sequences were more likely to continue possession and finish successfully. Switching from making a pass from an outfield play from the DF zone to the MD or MO meant a decrease in the probability of continuing possession or finishing successfully. In relation to PLO, the zones furthest from the own goal (CE, MO and OF), compared to DF, showed greater probabilities of not succeeding than of continuing possession. All the zones furthest from the goalkeeper reported higher odds of success than non-success.

## DISCUSSION

The aim of this study was threefold: to analyse how goalkeeper’s distributions are produced in Women Spanish La Liga in terms of habitual practices, incidence, and efficiency, to identify KPI’s and to check the power predictive of these KPI’s. Here, the average number of goalkeeper’s distribution was 28.9 per game which is similar to previous observations in men’s [24, 25] and women’s football [6] which analyzed the goalkeeper offensive actions.

Distributions from the goalkeeper, resulting in goals scored (0.4%) and attempts (2.2%) were low, with 79.4% of offensive sequences ending unsuccessfully, with the goalkeeper losing possession 32.5% of the time. These values are slightly lower than those reported by Iván-Baragaño et al. [26] who indicate that the possession of female teams end with no success in 75% of the occasions, 9% ended in a shot, and 1.1% with a goal. Maneiro et al. [27] also report slightly higher result, with 69% of ball possession ending unsuccessfully, 2.1% ending in a goal and 11.2% ending in shot. Although we must consider that in both studies the offensive sequences analyzed were initiated by all the players, not only the goalkeepers. Sainz de Baranda et al. [6] found that 37.7% of the attacks started by the goalkeeper lead to a loss of possession. Despite the matches corresponding to the FIFA Women’s World Cup being analyzed in these studies, the goalkeeper’s offensive efficiency was like that of the outfield players.

The bivariate analysis shows that the best teams, win and winning teams had more successful distributions than the rest of the teams. Surprisingly, two factors that, a priori, could influence the outcome of the offensive sequences, such as match location and defensive pressure, have not shown significant differences. Match location has been identified as a key factor in the offensive performance of women’s teams [28] and defensive pressure over the goalkeeper, could lead to an increase in the number of errors, however, these circumstance did not led to a decrease in the goalkeeper’s offensive effectiveness which coincides with work in men’s football [29]. A possible explanation is an increase in the goalkeepers technical-tactic skill level with the feet, who are increasingly used to solve one-on-one offensive situations. Another possible explanation, is that opposing team only put pressure on the goalkeeper, but did not close the passing lines to their teammates, resulting on this type of pressure being ineffective. However, as pressure of teammates was not measured here, it is necessary to continue investigating these situations to understand the influence of these two factors on the offensive performance of goalkeepers and included the pressure over the rest of offensive player.

Indirect distributions were more effective in comparison to direct distributions. Logically, indirect distributions involve short passes to nearby players hence will be more effective in comparison to long passes to more distant players using a direct distribution. The probability of losing possession of the ball during direct distributions is higher, due to lower precision of the pass and greater difficulty of the reception with defensive players having increased time to decide and act to intercept or win the ball. In addition, a long pass by a goalkeeper in women’s football does not usually exceed the midfield zone, so a second play against will be a disadvantage for the team as the defensive line faces the team’s midfield and forward lines.

The greatest offensive participation of the goalkeeper consisted of giving continuity to the game, giving support to their teammates, and starting the game after an offensive transition. In addition, these interventions have shown greater effectiveness than static actions. Similarly, Sainz de Baranda et al. [6] indicated that the kick pass was most frequently used offensive action. This is a fundamental cir-cumstance in today’s football to ensure possession of the ball is retained when the team has no chance of progressing forwards, with back passes to the goalkeeper ensuring possession of the ball and result in more passing options by increasing the available field of play space. This situation means that the offensive participation of the goalkeeper has increased considerably, as corroborated by the work of Sainz de Baranda et al. [6] who suggest the goalkeeper had become another outfield player to keep possession of the ball and offer new attacking possibilities.

The number of passes also turned out to be an indicator of the offensive performance of goalkeeper distributions. As happens with possessions without the intervention of the goalkeeper, possessions with a greater number of passes offer greater guarantees of success than those of short duration. Finally, pitch location of distribution has also been shown to be a key performance indicator in goalkeeper’s distributions. The goalkeeper’s distributions were more effective to the middle defensive zone and were less effective to the offensive zone. These can be explained, like indirect distributions as the goalkeeper’s short passes are to the areas closest to their goal and pose less risk than long passes to areas further away and with greater defensive density.

The multivariate analysis has allowed us to find five predictors of the outcome of the goalkeeper distributions (Team quality, Distribution type, Nº passes, Pitch zone of first pass by outfield play and Pitch location outcome). Being a team with a medium performance level offered 1.2 times more chances of being successful in offensive sequences in which the goalkeeper participates, supporting previous research that indicated the goalkeeper of higher level teams were more successful in distributions [6, 7, 30]. These results are likely explained by the higher technical-tactical level of the players of the best teams and by the tactical of these teams. The bottom teams usually have less tactical predisposition to start the offensive phase by playing the ball short.

Starting a goalkeeper distribution through Open play after transitions, that is, with a dynamic offensive transition, meant an increase of almost 3 times compared to doing it through a free kick. This result supports the idea of previous studies that indicate that transitions offer greater probabilities of offensive success than offensive sequences that start in a static way [29, 31, 32], due to the defensive imbalance of the rival team.

Number of passes also revealed as a good predictor, specifically, increasing the number of passes, increases the probability of success by more than 3 times, coinciding with the studies that indicate that possessions of longer duration are more effective offensively than those of short duration [14, 29, 31, 33, 34]. Here, the start of the offensive sequence is carried out from a position far from the opponent’s goal and, therefore, it will be necessary to make a minimum of passes to be able to take the ball towards the goal area.

The passes of an outfield player with the highest probability of success were from the defensive zone. Receiving passes from further away areas, slightly lowered the chances of success and of continuing possession of the ball. The explanation of these results may be the same as that indicated for the type distributions (greater distance of the pass, less precision, greater difficulty in reception, and greater defensive possibilities).

Finally, Pitch location of outcome was the best predictor of distribution outcome. However, this data does not provide very relevant information, because it is obvious that the closer the offensive sequence ends to the rival goal, the greater the chances of success it will have, as the highest percentage of goals and shots are made from areas close to the rival goal [22].

The results of this research allow us to know the usual practices of the goalkeeper’s distributions in elite women’s football, their key performance indicators and how to modify these indicators to increase their effectiveness. This information can be used to design training programs with specific loads for goalkeepers and to try to promote the reproduction of behaviors that favor success and avoid less favorable behaviors in these game situations. In addition, this information could help coaches to select the strategy to execute this type of game situations and to justify their decisions to their players.

This study does includes some limitations. Firstly, only one national league has been analyzed, so the results will only be extrapolated to this population. In addition, since this is a league with large differences in team quality between the participating teams, the quality of opposition in the analyzed games could be a variable that affects the outcome of the analyzed actions. For this reason, future research approaches should be directed towards the study of different national leagues and/or national team championships to obtain a more homogeneous sample of matches. Lastly, this aspect could help to improve the predictive power of the statistical models proposed. In this work we have only analyzed the pressure exerted on the goalkeeper, but not on the rest of the field players, nor have we analyzed the passing lanes offered by the outfield players, when the goalkeeper had possession, nor the spatial layout of the outfield players, both from the observed team and from the opponent. Therefore, future work could take these issues into consideration when designing the observation instrument, since it could help explain some of the results obtained. Considering these results, the technical staff should train the goalkeeper-initiated offensive dynamic transitions with the characteristics that show the results to increase performance in these game situations.

## CONCLUSIONS

According to the results obtain in the current research, it can be concluded that the offensive effectiveness of the goalkeepers is like that all of outfield players, since the success of the exit of offensive sequences with goalkeeper’s participation is like that of outfield players. The greatest offensive participation of the goalkeeper is carried out to give continuity to the game and in dynamic offensive transitions, the latter being the ones that offer a greater probability of success. To increase success in these game situations, passes to the goalkeeper should be from the defensive zone and the goalkeeper should send the ball to the near zones by means of a short pass and the offensive sequence should be built with 3–6 passes. Direct distributions by the goalkeeper, by means of long passes to areas away from the goal, frequently end with a loss of possession by the goalkeeper. Therefore, the goalkeeper plays an important role in ensuring possession of the ball and giving continuity to the game and in dynamic offensive transitions.
